# Assessment of Various Multimodal Fusion Approaches Using Synthetic Aperture Radar (SAR) and Electro-Optical (EO) Imagery for Vehicle Classification via Neural Networks †

**DOI:** 10.3390/s23042207

**Published:** 2023-02-16

**Authors:** Ram M. Narayanan, Noah S. Wood, Benjamin P. Lewis

**Affiliations:** 1 Department of Electrical Engineering, The Pennsylvania State University, University Park, State College, PA 16802, USA; 2 Multi-Sensing Knowledge Branch, AFRL/RYAP, U.S. Air Force Research Laboratory, Wright-Patterson AFB, Dayton, OH 45433, USA

**Keywords:** convolutional neural network, data fusion, decision level fusion, electro-optical, feature level fusion, machine learning, ResNet, SAMPLE dataset, synthetic aperture radar

## Abstract

Multimodal fusion approaches that combine data from dissimilar sensors can better exploit human-like reasoning and strategies for situational awareness. The performance of a six-layer convolutional neural network (CNN) and an 18-layer ResNet architecture are compared for a variety of fusion methods using synthetic aperture radar (SAR) and electro-optical (EO) imagery to classify military targets. The dataset used is the Synthetic and Measured Paired Labeled Experiment (SAMPLE) dataset, using both original measured SAR data and synthetic EO data. We compare the classification performance of both networks using the data modalities individually, feature level fusion, decision level fusion, and using a novel fusion method based on the three RGB-input channels of a residual neural network (ResNet). In the proposed input channel fusion method, the SAR and the EO imagery are separately fed to each of the three input channels, while the third channel is fed a zero vector. It is found that the input channel fusion method using ResNet was able to consistently perform to a higher classification accuracy in every equivalent scenario.

## 1. Introduction

Machine learning is an important component of the growing field of data science. Using statistical methods, algorithms are trained to make classifications or predictions and to uncover key insights in data mining projects, which subsequently drive decision-making in several application areas. Deep learning is quite popular and it seeks to extract the representative features of inputs in order to identify unique patterns in the data. Machine learning is widely used in image processing and computer vision, with applications in defense, commercial, and biomedical fields. The convolutional neural network, or CNN, has emerged as a pivotal ingredient in machine learning applications in image processing. CNNs are networks comprised of many successive convolutional filters which are trained via automated learning.

Target recognition in high-resolution imagery containing clutter and noise is a deep-rooted challenge. The development of approaches for automatic target recognition (ATR) has been aided by machine learning, merging concepts of image processing and classification with traditional radar processing techniques. ATR requires two steps: first, detecting possible targets in a large sensor dataset, and then classifying (i.e., identifying) those detected targets. In this paper, we primarily address the task of target classification.

Data fusion is an important concept for decision-making in target recognition. Significant attention has been given to data fusion techniques to optimize decision-making in systems of multiple sensors via machine learning [1]. Data fusion seeks to achieve a more accurate decision output than can be made by any individual sensor or data modality alone. The challenge of fusing synthetic aperture radar (SAR) and electro-optical (EO) imagery is of particular interest to the defense community due to those sensors’ data availability and orthogonal nature vis-à-vis data.

SAR is an active remote sensing technique that utilizes the movement of the platform to synthesize a large antenna aperture to improve azimuthal resolution. Data and imagery produced by SAR and EO sensors have both similarities and differences which make them a unique combination for fusion [2]. First, they exist in highly separated and distinct parts of the electromagnetic (EM) spectrum; for the images discussed herein, SAR operates in the X-band, from 8–12 GHz, and EO in the visible band, from 400–790 THz, corresponding to wavelengths of 2.5–3.75 cm for SAR, and 380–750 nm for EO. Consequently, EM waves interact differently with object features for SAR and EO sensors, leading to variations in image resolution, reflection properties, shadowing, etc. Thus, an algorithm or process designed for one modality may not perform well for the other.

This paper provides a comparative example of several fusion methods on SAR and EO data. Each data modality is first processed separately to provide a baseline. Then, fusion approaches using decision-level fusion, feature-level fusion, and a novel fusion method using red/green/blue RGB-input channels for an image processing network, are tested. The channel-based fusion is an off-the-shelf implementation of feature-level fusion. The classification performances of each method are compared when implemented using an 18-layer residual neural network, or ResNet, and a simpler six-layer custom-programmed network. Each network type is repeatedly tested to gauge the variability between runs and the typical distribution of possible outcome accuracies.

We emphasize that the goal of this paper is not to achieve the utmost accuracy, nor to inform the reader as to which of these particular networks is “best.” We are seeking to compare network convergence and performance across network sizes and fusion methods. In real-world scenarios, the network with the best performing accuracy and speed would be chosen, based on the situational technical requirements. It is impossible to declare, for certain, a single winner among the compared networks, because they are not in competition. Instead, we seek to examine the difference in how these networks and fusion methods train and classify.

Section 2 introduces the design and background of the experiments, including the network structures, machine learning variability, training, and testing structures, and the datasets used. Section 3 details the various fusion methods compared. Specific methods and learning parameters are discussed in Section 4. Section 5 discusses the results of the numerical experiments, with Section 6 providing concluding remarks.

## 2. Experimental Background and Design

### 2.1. Brief Description of Networks Tested

The primary goal of this paper is to assess the abilities of neural networks to classify targets based on SAR and EO imagery, utilizing different methods for data fusion. To satisfy this goal, two levels of networks and three different types of fusion were selected. The intention was to compare how basic implementations of fusion would perform relative to each other. The networks and fusion methods are detailed in the following sections.

**Figure 1:**
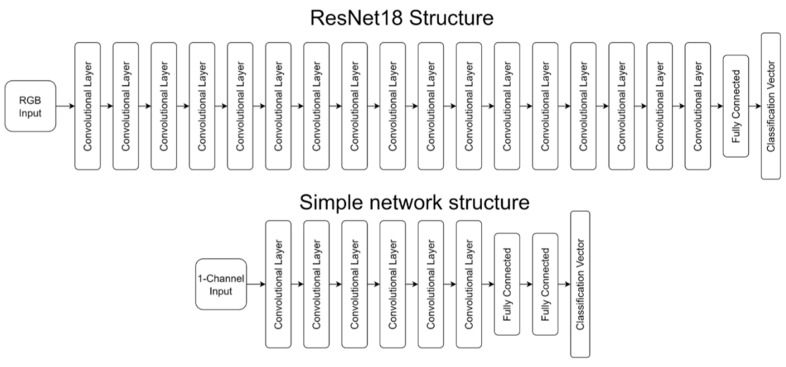
Network structures.

The first type of network was the Python PyTorch package implementation of ResNet [3], specifically ResNet18, the smallest available size. ResNet18, shown in Figure 1, is an 18-layer residual deep neural network containing shortcut connections that allow some layers to be passed over. ResNets are image-processing networks requiring a three-channel RGB input. PyTorch also makes available 34-, 50-, 101-, and 152-layer ResNets. The 50-layer version, ResNet50, is used for comparison purposes later in this paper.

The second network type was a simpler custom-built network, containing six convolutional layers followed by two fully connected layers, also shown in Figure 1. This network is hereinafter referred to as the “simple net” or “Simple Net.” The simple net has a 1-channel input, meaning it takes in a single-channel image, whereas the ResNet has a three-channel input for RGB imagery, and is mainly studied for classification accuracy comparison vis-à-vis ResNet.

The MSTAR and SAMPLE datasets (discussed in Section 2.5) were separated into individual classes and prelabeled for supervised learning. Supervised learning is the process of training a neural network where it is given both an image and its class label, so it can draw conclusions regarding the shared features of a given class. The alternative is semi-supervised or unsupervised learning, which requires the network to identify features and find clusters of similar images without the help of class labels [4].

The general goal of the experiments conducted in this paper was to compare the networks’ and fusion methods’ relative abilities to fuse and disperse data in the form of SAR and EO imagery. Each network implemented the fusion methods (single data type, feature level, decision level, and RGB-channel in the case of the ResNet) to serve as a comparison of their relative effectiveness in the form of classification accuracy.

### 2.2. Variability and Multiple Iterations

Each experiment in this paper was repeated 50 times in order to provide descriptive statistics. In general, the same network model with the same data will not converge to an identical result every time it is trained. Some of the reasons for this variability are discussed below. To characterize the relative variability of the methods, the distribution of results of each method over the many iterations can be collected and compared.

An important issue is that of the “replication crisis” or “reproducibility crisis” [5]. Researchers found that a massive proportion of published research results was completely irreproducible subsequently when attempted by other researchers, who would produce entirely different results compared to the original. This issue spawned the field of “metascience,” which purports to strengthen the scientific body of knowledge, avoiding issues of reproducibility through statistics and meta-analysis of methods and results [6].

While the above issue was typically found in the context of natural science fields, a similar phenomenon has been sometimes observed in machine learning research [7]. There are multiple causes and explanations that contribute in varying degrees to this issue in the machine learning context. Firstly, there are many different esoteric parameters such as learning rate, batch size, and epochs of training that will have a significant influence on the final result while going underreported. Secondly, there is an even more fundamental level of randomness in the form of regression algorithms such as stochastic gradient descent, one of the most common forms of training for neural networks.

Given a certain dataset and training parameters, a particular network may fail to converge to a suitable solution half of the time it is trained while converging to a reasonable model in the other half. If a researcher runs and publishes results with a successfully converged model, a replicating researcher may very easily find themselves with a nonconvergent model if proper documentation is not provided [8]. There is also the impetus among researchers to publish their code and models [9], although this is not possible for those working on sensitive or proprietary research.

With the variability of machine learning in mind and the importance of reproducibility as a whole, we set out in examining ways of analyzing and visualizing variability in our results. One such way we found was through collecting and comparing results from many iterations of the same experiment and through tools such as violin plots [10], which expand on a typical box-and-whisker distribution plot with local density estimates.

There are multiple sources of variability in the machine learning process, which can lead to difficulty in replication. While those elements can be fixed for a certain problem, there are purely stochastic variables that will always cause some variability. Those include the stochastic gradient descent process, random filter weights initialization, etc. Different networks, datasets, and fusion methods will experience the effects of this to different extents. To help better understand the effects of these stochastic elements, each experiment (training a given network implementing a fusion type) was repeatedly run to gather a distribution of results. Through many iterations, the goal was to gather enough data to compare the likely outcomes of the networks and the positive or negative influences data fusion could have on the outcomes.

In the real world, instances and outcomes with the best accuracy could be selected, and the worst performers simply discarded. However, our goal was not to simply find the most accurate network, but rather to compare the likely distribution for a given result. In many cases, there are limitations on how many times a model can be trained, either due to a much larger dataset, more complicated models, or a lack of computational resources and time. Therefore, an understanding of the variability and potential ways to encourage more consistent higher accuracy results would be very useful.

### 2.3. Training Length

In machine learning, feeding the training set to the network one time is referred to as a single ‘epoch’ of training. One epoch is typically not sufficient for a network to realize its full potential, so networks are trained over multiple epochs, passing over the same training data multiple times. However, by forcing the network to repeatedly train on the same data too many times, the network risks overfitting and degrading the testing accuracy. Overfitting occurs when a network becomes so well adapted to the training set that it no longer represents a generalized solution including the testing set. Prevention of overfitting is a crucial part of the training process, and in most cases, a subset of the training set is set aside as validation data.

There exist several ways in which the training process may be terminated [11]. If an application requires an algorithm to perform above an accuracy threshold, training may be cut off as soon as that threshold is met. Another method is to observe the training loss (or the rate of improvement in accuracy being made by the network as it trains), and when loss becomes sufficiently low, to end the training. For consistency, a fixed training length of 10 epochs was chosen in our work, as it was long enough that the networks’ accuracies had stabilized, but not too long that they began overfitting.

### 2.4. Training and Testing on Separate Elevations

State-of-the-art image recognition neural networks have been shown to achieve extremely high classification accuracies when tested on images similar to the training set. Such results show that given a fairly similar set of data, neural networks can provide extremely impressive performance.

While the performance of neural networks within their training conditions is laudable, we are also interested in how well a network may respond to inputs different from its training conditions. Due to the difficulty of gathering expansive datasets covering all possible operating conditions, networks in the field may be expected to perform on data somewhat similar to their dataset, but to which they have no prior exposure.

Instead of implementing continually learning systems, our work focused on systems being asked to make in-the-moment identification decisions on a closed number of immutable classes, without the chance to spend time identifying new clusters.

As such, our experiments used the following structure: train on a set of lower elevation data (with an altitude angle of 14 or 15 degrees above ground), validate on data of that same elevation, and test on higher elevation data (17 degrees altitude angle). By training and testing under slightly different conditions, we aimed to test the networks’ robustness to change; although there is a penalty incurred on the overall accuracy. However, we have emphasized that we care less about the final accuracy and more about comparing the relative performance of these networks and fusion methods. The absolute performance of such networks has been previously proven and explored in existing works.

Obviously, this example is not indicative of a likely reality, as a real-world network would have access to training data at many elevations. However, it is still a useful lens through which to examine the performance degradation of networks when asked to act outside their trained conditions. It is similarly useful for questions of gathering datasets and creating new synthetic data, such as in the case of SAMPLE, where it is desirable to know the required resolution between image angles in the training space, as well as the necessary level of similarity to prior images.

One way in which we can compare in-training-conditions performance to out-of-training-conditions performance is via the validation set accuracy to the testing set accuracy. The validation images are a subset of 15% of the available training images that were separated and not used in the training process. As the validation images are at the same elevation as the training images, although at different azimuth angles, a network’s accuracy on them can be used to view its accuracy on images inside of the training conditions.

### 2.5. Datasets: MSTAR and SAMPLE

As the primary goal of this paper was the fusion of EO and SAR imagery, to this end, a suitable source of realistic and fusible SAR and EO data was required. One realistic scenario is one in which SAR and EO imagery of ground targets are available from the same elevation and azimuth angle. This provides an analog to the real-world scenario where a SAR and EO sensor has been mounted to the same airborne platform and taking aerial data of vehicles far away on the ground, thereby producing imagery from the same angles.

To provide a framework to compare fusion methods, the SAMPLE dataset [12] was used to train and test our neural networks (and fusion methods they implemented) to classify the 10 military targets contained in the database. The SAR data and supplementary EO data of the SAMPLE dataset at a resolution of 128 × 128 pixels were used. The SAMPLE dataset is an augmented version of the MSTAR dataset [13], a commonly used set consisting of SAR images of a variety of military targets. These original images were collected by taking manual SAR measurements of scale−model vehicles at a variety of azimuth and elevation angles; however, the degree of spacing between images is somewhat high.

The MSTAR and SAMPLE datasets consist of ten labeled classes of military vehicles. The class names correspond to the model of the vehicle included in the class, consisting of 2S1, BMP2, BTR70, M1, M2, M35, M548, M60, T72, and ZSU23-4. Of these, the 2S1, BMP2, M1, M60, and T72 are different types of tracked tanks (running on continuous treads, as opposed to wheels). The M2 is a tracked infantry fighting vehicle, similar to a small, light tank. The M548 is a tracked cargo carrier, and the ZSU23-4 is a tracked anti-aircraft vehicle. The BTR70 is a wheeled armored personnel carrier, and the M35 is a wheeled cargo truck.

Blasch et al. compiled a review of machine learning projects utilizing MSTAR in [14]. The MSTAR dataset has been used as the basis for many experiments in artificial intelligence and machine learning for the field of automatic target recognition. Numerous authors have used MSTAR as a training and testing set for the classification of targets using neural networks. Specifically, Morgan [15] and Wang et al. [16] showed that CNNs were able to give a high degree of classification accuracy on the 10 classes of the MSTAR dataset.

**Figure 2:**
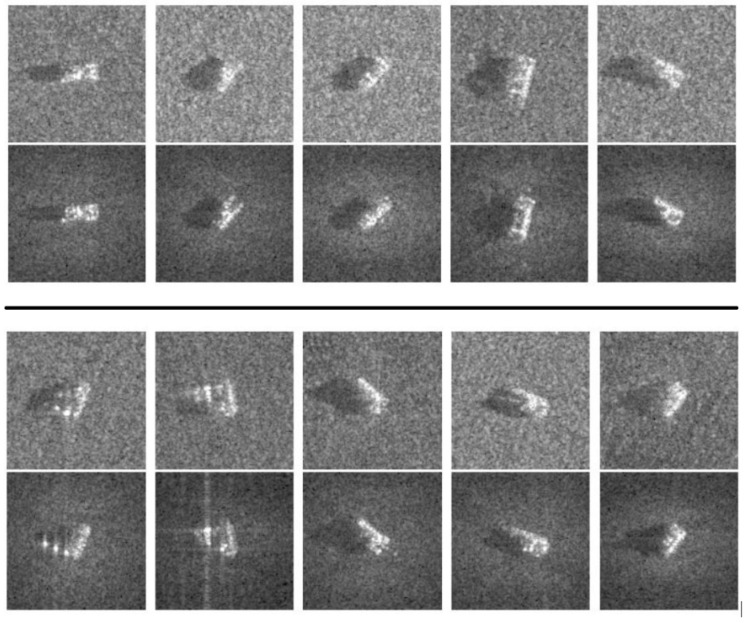
Images from each class of SAMPLE (top) next to MSTAR (bottom); 2S1, BMP2, BTR70, M1, M2 (above), and M35, M548, M60, T72, ZSU23-4 (below). Reproduced with permission from [12]. 2019, SPIE.

The authors of the SAMPLE dataset set out to expand the number of images using synthetic data based on the original images. Because there are many possible parameters, target configurations, and external conditions which widely affect the SAR images, it is reasonable to say that the operating condition space (i.e., the combination of possible configurations) of synthetic aperture radar is extremely large. To help fill in the gaps in the imagery of operating conditions that do not exist in or are lacking in the original MSTAR database, the SAMPLE database was created by expanding MSTAR with well-matched synthetic data. Figure 2 compares the original MSTAR imagery to the generated equivalent in SAMPLE.

**Figure 3:**
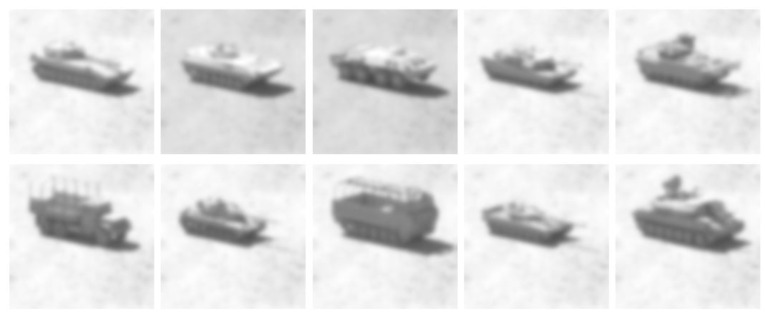
Examples of EO data from each class (class order remains the same). Reproduced with permission from [17]. 2022, SPIE.

As part of the creation of SAMPLE, additional synthetic electro-optical imagery was created to enable the testing of fusion methods. An example of these images is shown in Figure 3. These synthetic images are based on the same CAD models that the SAR imagery was generated from. The CAD models were carefully based on available metadata on the original MSTAR models. As the relevant radar frequencies have wavelengths on the centimeter scale, the models were made to ensure they were accurate down to that same level.

The authors of SAMPLE focused on a subset of 10 of the original targets used in the MSTAR dataset. Material properties for the models were gathered from MSTAR notes as well as the best knowledge as to the material of the parts. For purposes of image generation, the models were placed on stochastic rough surfaces to generate appropriate background speckles (although at the time of writing, it was not ideally matched to the background speckle of the original MSTAR database). Synthetic phase history was extracted from the metadata of the original MSTAR set. Using the phase history and the original angles, ranges, resolutions, and bandwidth, the points at which the images would be taken were constructed, in effect creating an artificial flightpath around the target for use in the SAR imaging programs. The authors compared the synthetic images to the original dataset using neural networks and were able to show improvements over the previous literature. One of the main reasons that the SAMPLE dataset was created was to evaluate networks’ ability to generalize from training synthetic-only data to testing on real-world data. This particular challenge is a representation of the fact that the vast majority of operating conditions cannot be achieved in a single dataset, meaning it is difficult to train a network for every possible set of operating conditions it might encounter.

## 3. Comparison of Fusion Methods

Data fusion is a broad-reaching term, often divided into three structural categories: data level, feature level, and decision level. These are also sometimes defined as early, middle, and late fusion, or low, middle, and high fusion, depending on the usage and specific implementation. For our purposes, data fusion is being used to combine input images from two sensors in the form of matched SAR and EO imagery, with the goal of improving the classification of labeled data using neural networks. Tong et al. [18] provided a review of data fusion techniques for machine learning.

Three different fusion experiments were implemented with the simple custom-defined neural network and the ResNet: SAR and EO individually (no fusion), a layer concatenation (feature level fusion), and a vote (decision level fusion). Additionally, the ResNet’s three−channel structure was used to implement a fusion technique where EO images and SAR images were fed into two of the channels (functionally, another form of feature-level fusion at an earlier level than our bespoke method). These fusion methods were selected to evaluate how a basic implementation of each type at the middle and late levels would perform relative to each other, and to networks with only one sensor.

### 3.1. SAR and EO Standalone

Herein, the performance of the networks is evaluated on SAR and EO data individually. Only SAR data are used to train the SAR network, and only EO data are used to train the EO network. This provides a baseline performance of what each modality is capable of on its own, thus the standalone experiments are representative of systems without fusion. This is how image processing neural networks are typically used, where a single type of data (normally images) from a single dataset is used for training and testing.

### 3.2. Decision Fusion

Decision-level fusion refers to fusion methods taking place after the final classification layer of networks. There are many explored methods for fusing the outputs of multiple classifiers, including a variety of different voting methods. A comprehensive survey of decision-level fusion techniques in automatic target recognition is provided in [19].

In this experiment, the results of individual networks were combined via a very simple form of decision fusion. The classification vectors of each network were extracted and averaged, a simple way of allowing multiple networks/sensors to vote on the class of input. The class scores from the output of the final fully connected layer were taken and averaged, meaning the SAR-only network and the EO-only network separately made their classification decisions before the scores were aggregated. The two networks were trained on individual modalities, as in the standalone network experiment. Only the output decision vectors of the networks were combined, meaning no structural interaction or interference took place between the two networks.

An advantage offered by decision fusion is its ability to easily handle many different data and network types. The channel fusion method discussed below allows only imagery inputs, and feature-level fusion would require significant retooling to accommodate other data types. Decision fusion, however, can easily incorporate the outputs of many types of sensors into the voting process without the need for a significant reworking of any network structures.

### 3.3. Feature Level Fusion

There are many configurations and approaches to middle fusion popular in the fields of image processing and discrimination. Our implementation here is to combine information at the output of the final feature layer, where the networks have produced their respective final feature vectors. The term feature vector is used here to describe the layer preceding the fully connected layers and decision layer. This project focused on concatenating those feature layers of two networks that processed EO and SAR data separately.

**Figure 4:**
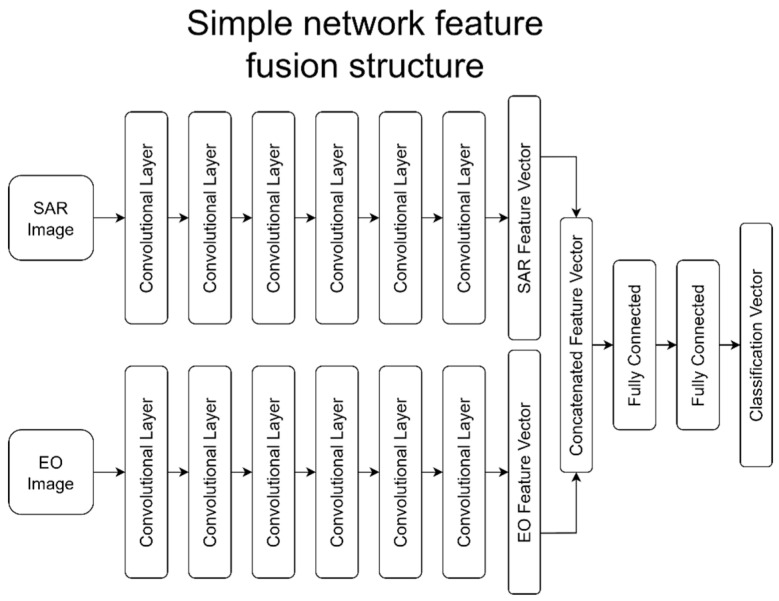
Examples of EO data from each class (class order remains the same).

Feature level fusion was used to fuse the respective feature vectors from the individual SAR and EO networks. In this implementation, the final level of feature vectors from a SAR and EO network were concatenated, then sent through a standard multi-layer fully-connect network to make a single classification vector. In effect, the SAR and EO networks ran in parallel until their final stages, where they were fused and run through the structure of a single network to the classification layer. Figure 4 shows a graphical depiction of the feature-level fusion structure for the simple network.

### 3.4. RGB Input Channel Fusion

The channel-based fusion presented here used the multiple input channels of an image-processing-oriented neural network to accept multiple data modalities. The ResNet networks used in this implementation expect to see a three-channel input, typically the red, green, and blue channels of color images. The previously discussed networks used SAR or EO images converted to 3-channel grayscale, with equal values in each of the RGB channels. In the channel fusion scheme, EO data was fed into one channel, SAR data was fed into the second channel, and an empty vector was fed into the third channel. If a third orthogonal input sensor were to become available, it would be very easy to incorporate this into the third channel; as only two were available here, SAR and EO, only the first two channels were used.

This approach requires that the SAR images and EO images have the same resolution, which could lead to some accidental data loss if proper scaling is not conducted to match the image resolutions. Speculatively, unmatched data may cause more issues with this fusion method than others, as input channels are more intrinsic to the structure of the network. On the other hand, providing two disparate images to the network may improve the total level of information available for decision-making, thus improving accuracy.

It may be tempting to compare this method to data-level or early fusion methods, as the operations occur at the input to the network. Those are methods that fuse at the data or pixel level; the fusion here may seem to be performed at such a high level, by fusing the multiple data types into one three-channel image. In reality, the input-channel fusion may be more accurately described as a form of feature fusion. The SAR and EO data are not being fused together directly; instead, the neural network’s predefined structure is used as an algorithm to fuse at the feature level, albeit an earlier feature level than the previously discussed bespoke feature level concatenation we implemented.

## 4. Analysis Methods

The structure of the experiments was to train networks on low-elevation angle data and test on high-elevation angle data. Each class in the SAMPLE dataset had slightly different available elevations, so the training data consisted of any available samples below 17 degrees, while the testing data consisted of 17-degree elevation data. In the case of multiple networks being compared or combined, SAR and EO input data were as closely matched in elevation and azimuth as possible.

This meant that training and testing take place under slightly different operating conditions, allowing observation of how well the networks performed outside of their training conditions. This was carried out to help probe the networks’ flexibility under changing operating conditions, where the real-world operation may be happening under conditions dissimilar to training conditions.

All networks and fusion methods were implemented as Python scripts using the PyTorch machine learning package [20]. Each network and fusion type was created as a separate callable function which returned the relevant results of a single iteration of the experiment. A central script handled running multiple instances of each experiment, as well as saving the data and computing the averaging results.

The networks were trained and tested on synthetic data from SAMPLE and corresponding synthetic EO images. The dataset provided 4357 synthetic SAR images and 4357 matching synthetic EO images for training (referred to here as an image pair). Of the 4357 pairs of images, 15% were split off for use as a validation set. Following the validation split, that meant there are 3704 pairs of training images and 653 validation pairs. There were 2693 high elevation synthetic SAR and EO image pairs available for testing. The order of the training images was randomized to ensure networks did not fit one particular class, a common practice in neural network training.

All networks were trained with the same data over 10 epochs, to ensure all networks were able to maximize their performance on the training data. In addition to the testing accuracy, the loss, training, and validation accuracies were also recorded after each of the 10 training epochs. In typical machine learning problems, the loss and validation accuracy are two of the key metrics to ensure the network is not overfitting (no significant overfitting was observed in the space of 10 epochs for the experiments discussed here).

After each epoch, the networks were switched to their testing mode, and classified the testing data in their current state. The networks’ testing mode freezes the training of the network and prevents a network from continuing its training and improving its accuracy during a testing phase (the other mode of the networks is the training mode, in which the network is allowed to continue its normal optimization process). The goal of recording the networks’ testing-data accuracy after each epoch was to gain insight into how networks were likely to train over multiple epochs, with regards to understanding the inherent intrusion of the randomness of the machine learning process.

Since there is variability in performance when rerunning the same experiment, many iterations were run of each experiment type. This was conducted to facilitate comparisons of the distribution of accuracies and averaged values achieved over many instances. Each experiment was run at least 50 times, and after each iteration, the 10 epochs of training, validation, and testing accuracy were recorded. Violin plots after each experiment run confirmed that convergence was attained after 50 runs. The network states were also saved using PyTorch’s state dictionary function, allowing the networks to be reloaded later; this allowed for the later collection of confusion matrices for averaging, as well as allowing the standalone networks to be used in a deciding vote.

A learning rate of 0.001 was utilized along with a batch size of 32. Training parameters were kept the same between all experiments for consistency. The order of training images was randomized to ensure no overfitting occurred during training. When multiple networks were used in conjunction, the same randomization order was used on both the EO and SAR training sets to ensure that properly matched data was being fed to the networks.

Confusion matrices were generated using a custom function extending Python’s “scikit learn” confusion matrix class. Violin plots were generated using Python’s “seaborn” statistical data visualization package.

## 5. Results and Discussion

The results of each fusion method may be most easily compared by their overall classification accuracies. This is the simplest measure of their relative performance, a single−number summary of complex results. The final average classification accuracy on the testing data after 10 epochs of training is given in Table 1 for each experiment type, and each method is explored in more depth in the following sections.

As expected, there is a dichotomy in performance between the larger ResNet and the smaller simple net, with the ResNet performing better across all experiments by around a 10% margin in overall classification accuracy. For more detail, we can observe the violin plot of the final accuracy distributions of each method for the ResNet and the simple net. This provides an additional layer of intuition towards the probability density function of the final overall accuracy for a given experiment over a large number of iterations. In each plot, a central box plot is surrounded by an estimate of the density function.

**Figure 5:**
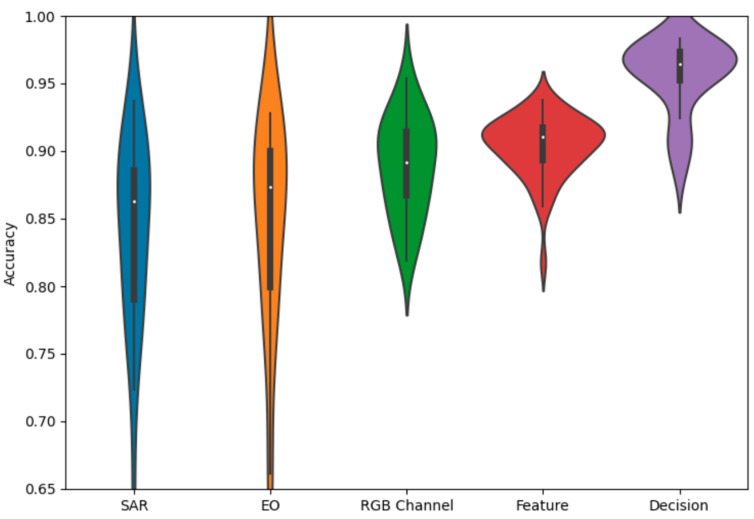
Accuracy distributions of fusion methods with ResNet18.

We also observe the overall comparison graph for the ResNet18 experiments in Figure 5. The SAR-only and EO-only are wider, with the fusion methods providing more compact distributions. All fusion methods provide some level of performance gain, with decision fusion providing the strongest results. The methods are also mostly approximately normally distributed, with a few low-performing outliers.

**Figure 6:**
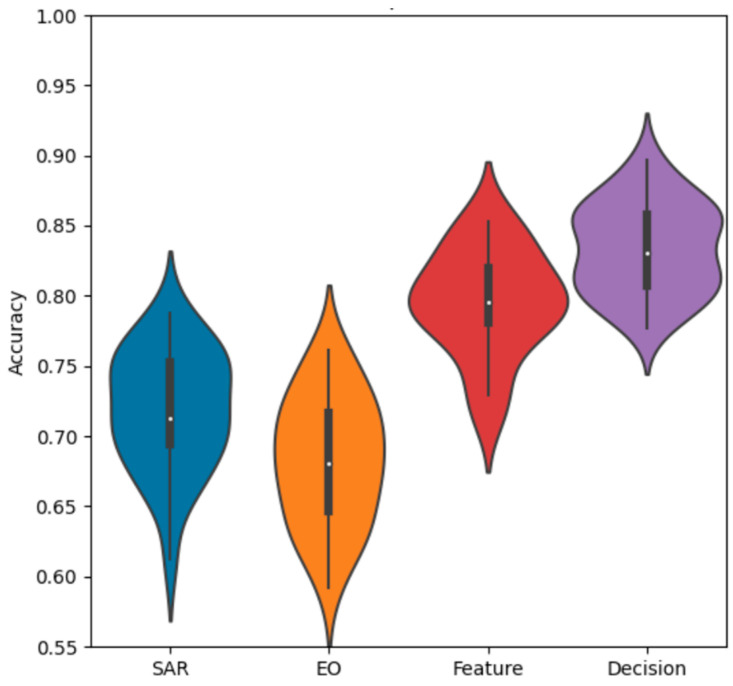
Accuracy distributions of fusion methods with a simple net.

The violin chart for the simple network, given in Figure 6, also reflects our overall expectation based on the numeric averages. The SAR-only network has a slightly higher distribution than the EO-only network, and both fusion methods provide a gain in performance over the single−modality networks. What is further noteworthy, is the difference in shape between the decision and feature fusion distributions, with the decision fusion having a slightly more bimodal shape compared to the more uniquely shaped feature fusion.

### 5.1. SAR-Only ResNet18

**Figure 7:**
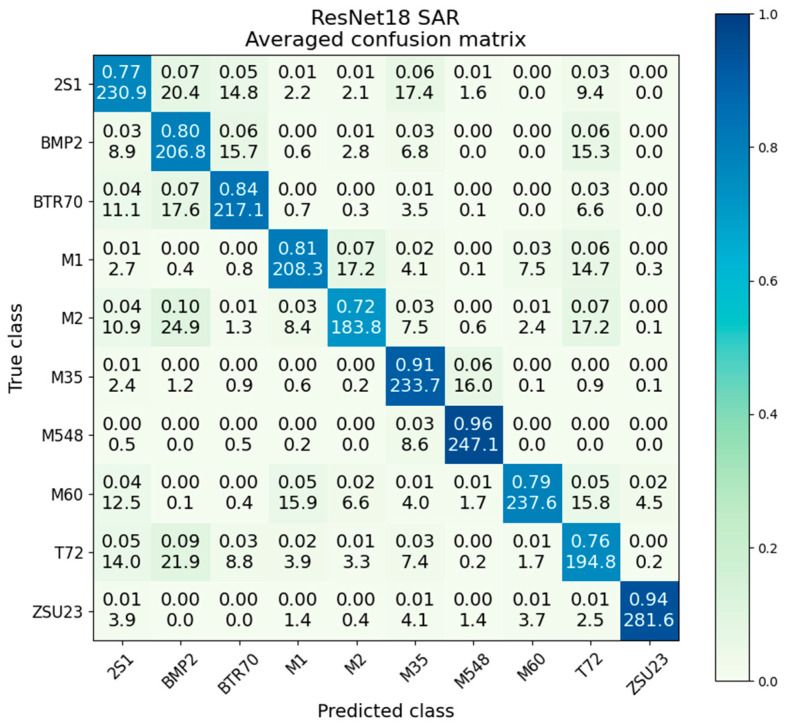
Averaged confusion matrix for ResNet18 on SAR data.

The SAR-only ResNet performs at an overall accuracy of 82.8%. The confusion matrix for the SAR-only ResNet18 is given in Figure 7. We observed generally acceptable performance across all classes, with the intraclass accuracies being between 72% and 96%. The mean and the intraclass (i.e., the central diagonal 2S1 to 2S1, BMP2 to BMP2, etc.) variance (Var) and standard deviation (SD) are also given in Table 2, taken from the generated variance and standard deviation matrices. The table is arranged from the highest variance on the left to the lowest variance on the right, with the 2S1 class having almost four times the variance of the ZSU23 class. In comparing to the intraclass accuracies, we see that the accuracy is roughly inversely related to the variance: higher variance classes tend towards a lower accuracy, and lower variance classes tend towards higher accuracies.

**Figure 8:**
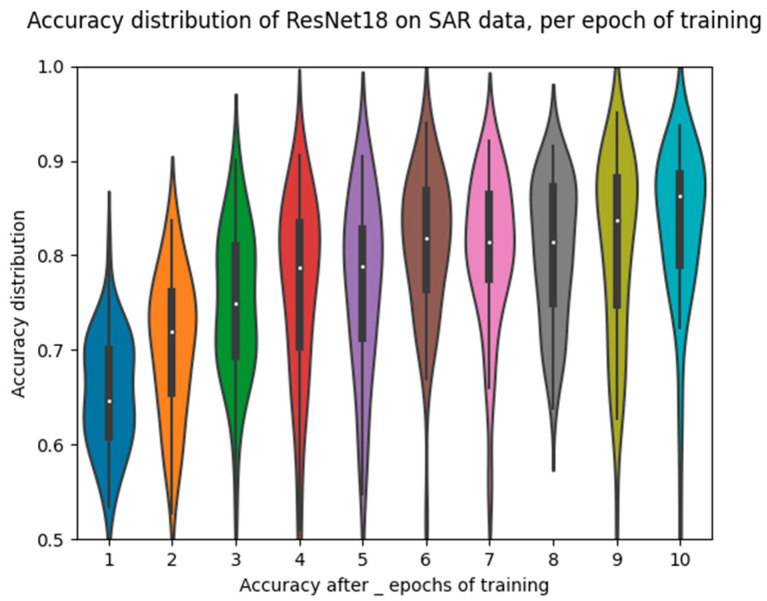
ResNet18 SAR-only accuracy distributions during training.

We can also observe the accuracy distribution after each epoch of training via the violin plot in Figure 8. We note here that the distribution remains at a similar density and spread across all epochs, which we will see later is characteristic of the standalone networks but less so for the fusion methods.

### 5.2. SAR-Only Simple Net

**Figure 9:**
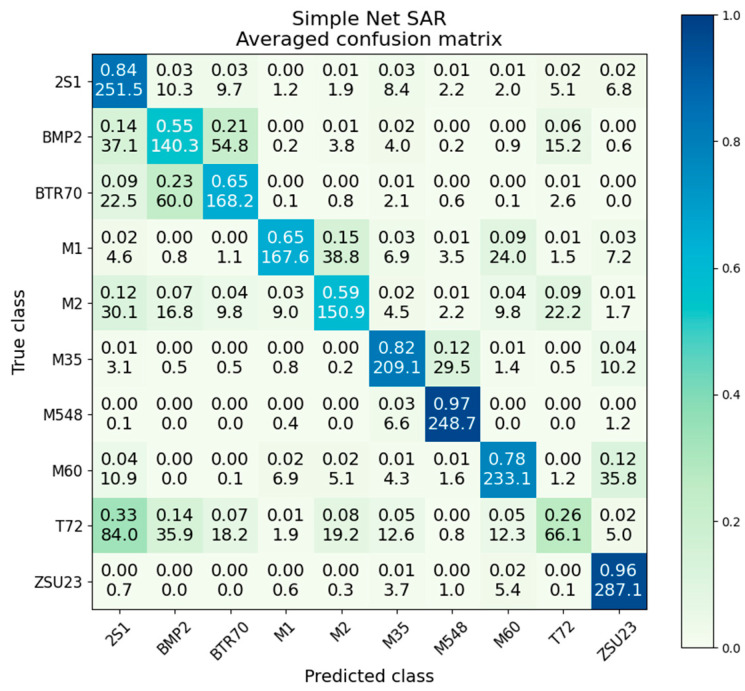
Averaged confusion matrix for simple net on SAR data.

The SAR-only simple net scores an overall classification accuracy of 71.8%, about 11% worse than the ResNet. The confusion matrix is given in Figure 9. Compared to the confusion matrix for the SAR-only ResNet, we see that there is significantly more interclass confusion, particularly between BMP2 and BTR70, with M1, M2, and especially T72 also performing quite poorly.

The only class where the simple net noticeably outperformed the ResNet was the 2S1 class, which scored 84% in the simple network and 77% in the ResNet. While this 7% performance gap is interesting, we also observed on the confusion matrix that the simple network overpredicts 2S1, predicting 33% of T72, 12% of M2, 9% of BTR70, and 15% of BMP2 as False Positives of the 2S1 class.

The intraclass variance and mean are given in Table 3, with the highest variance on the left and the lowest on the right. Again, we see the rough inverse correlation between variance/standard deviation and accuracy, where the lower a class’s accuracy, the more likely it is will have a high degree of variation between iterations. It is also observed that the ResNet and the simple net have similar ranges of variances/standard deviations. The ResNet has the lowest variance of 5.4 × 10^−3^ and a highest of 7.1 × 10^−2^; the simple net has the lowest variance of 1.6 × 10^−3^ and a highest of 6.7 × 10^−2^. The standard deviations are proportional to the variance.

**Figure 10:**
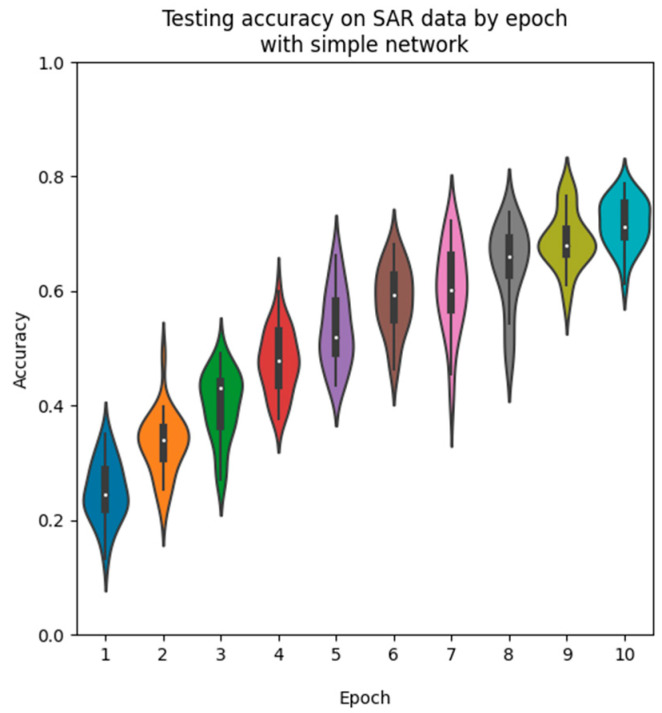
Simple net SAR-only accuracy distributions during training by epoch.

We also observe the testing accuracy after each epoch in Figure 10 and observe an even-more distinct pattern of training. The distributions form a very noticeable curve, which begins to taper off in the ninth and tenth epochs. The accuracy is also extremely low in the initial epochs, starting from a much lower accuracy and slowly working its way upwards. This shows the smaller size of the network requires more training to find a fit to the dataset, while the ResNet converges to near its final state much more quickly.

### 5.3. EO-Only ResNet18

**Figure 11:**
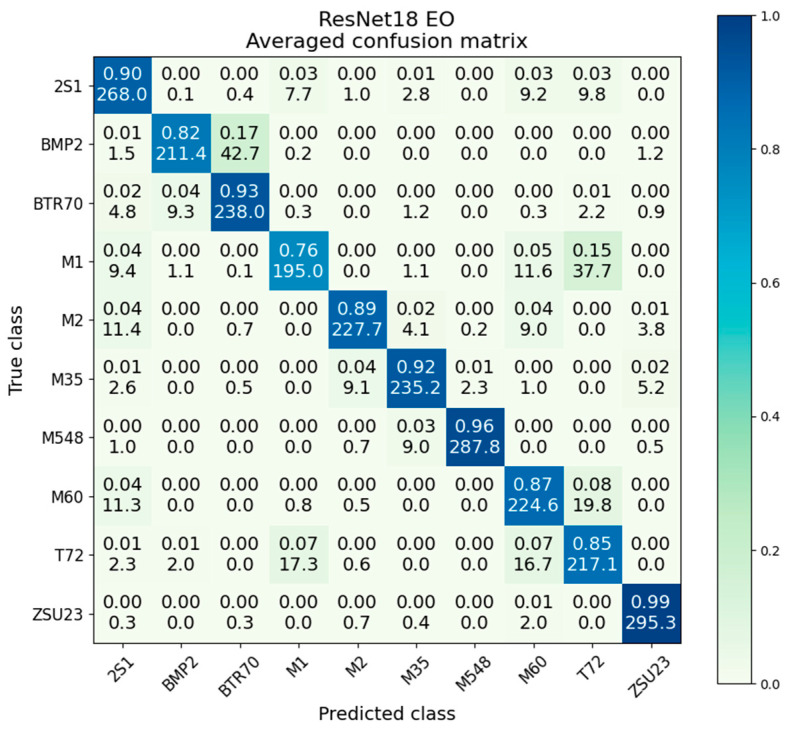
Averaged confusion matrix for ResNet18 on EO data.

The averaged confusion matrix for the EO-only ResNet is given in Figure 11. The worst performing class is M1, where we see the first instance of notable T72 False Negatives for the M1 class. We will see this particular confusion pattern between the M1 and T72 classes worsen during some ResNet fusion methods, so it is noteworthy that we first observe it here to a limited extent in the EO-only experiment. While 15% of M1 images were misidentified as T72, 7% of T72 images were misidentified as M1, showing the problem is worse for the M1 class than the T72 class.

The variance and standard deviation for EO-only ResNet are given in Table 4 and follows a similar pattern to the SAR-only results. Particularly, the general trend of lower accuracy classes having higher variance and vice−versa. Both modalities have their best performance on ZSU23 and strong performance on M548, but other classes are fairly divergent between the modes. For example, 2S1 is the second-lowest variance class in EO-only at 8.5 × 10^−3^ but has the highest variance in SAR-only at 7.1 × 10^−2^. The average variance is 2.7 × 10^−2^, compared to 3.2 × 10^−2^ with SAR-only data; this shows that, in addition to stronger accuracy, the EO ResNets will converge to a somewhat tighter distribution over many iterations.

### 5.4. EO-Only Simple Net

**Figure 12:**
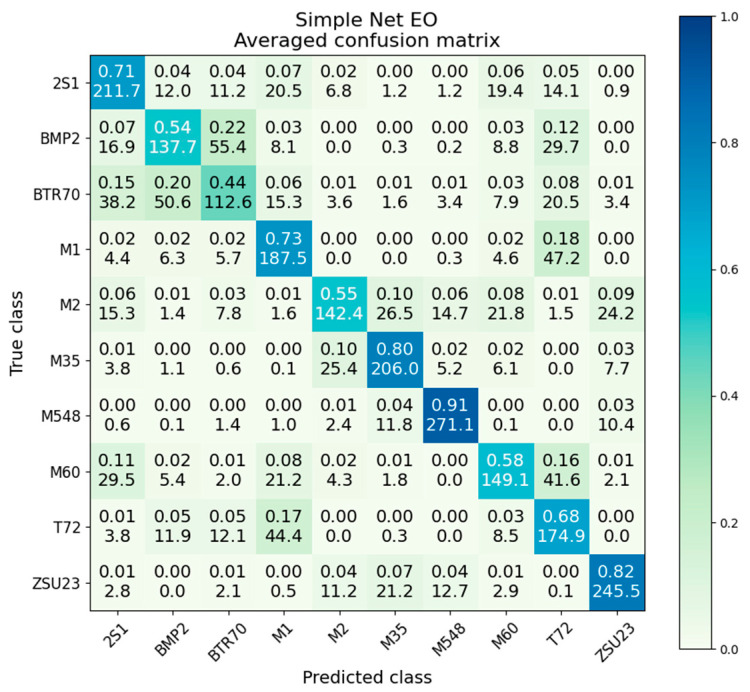
Averaged confusion matrix for simple net on EO data.

The confusion matrix for the EO-only simple net is given in Figure 12. There are a few notably poor-performing classes, with significant confusion between BMP2 and BTR70, and additional poor performance from M2 and M60. We note again the presence of M1/T72 confusion, showing it is present, to some degree, in all EO experiments, not just ResNet.

The variance and standard deviation for EO-only simple net are given in Table 5. The average variance is 2.02 × 10^−2^, compared to the SAR-only simple net at 2.45 × 10^−2^. While the overall accuracy is lower than SAR, the EO-only network still converges to have less overall variance. The T72 false negative square for the M1 class has a value of 2.0 × 10^−2^, half of the EO ResNet which scores 3.9 × 10^−2^.

**Figure 13:**
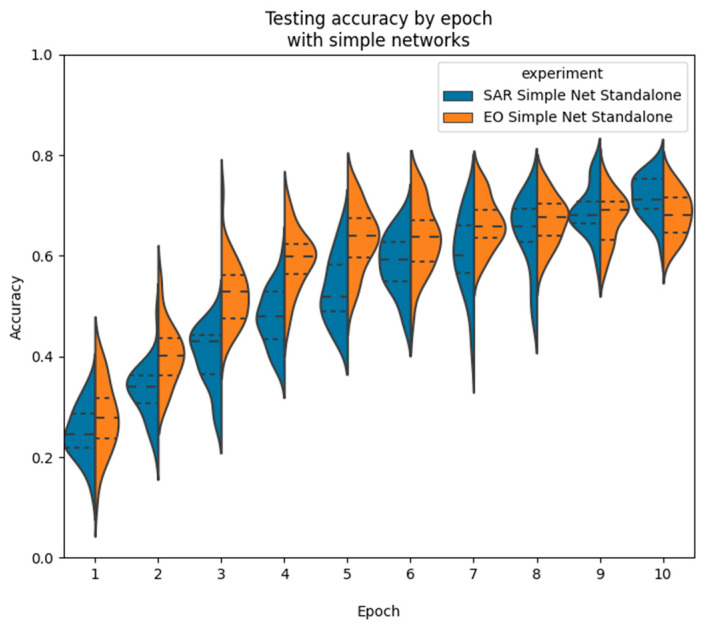
Simple net SAR vs. EO distributions during training by epoch.

The EO data follows the same general pattern during training as the SAR data. Accuracy distributions remain at a similar width throughout training, increasing for the first few epochs before mostly leveling off towards the end of 10 epochs. The simple net training pattern is plotted in orange next to the SAR data in blue in Figure 13. It is observed that the EO data begins at a similar accuracy, but converges more quickly to its final accuracy in the first few epochs, while the SAR network improves more slowly.

### 5.5. ResNet18 Decision Fusion

Decision fusion with ResNet scores an average accuracy of 95.6%, providing a strong improvement over either SAR (80.3%, a gain of 15.3%) or EO (82.8%, a gain of 12.8%) networks on their own. By involving the worse-performing SAR ResNet via the voting process, accuracy is able to be improved beyond the performance of EO ResNet alone. A significant performance gain is noted, with the result of decision fusion approaching near-perfect accuracy outside of the M60 and M1 classes.

**Figure 14:**
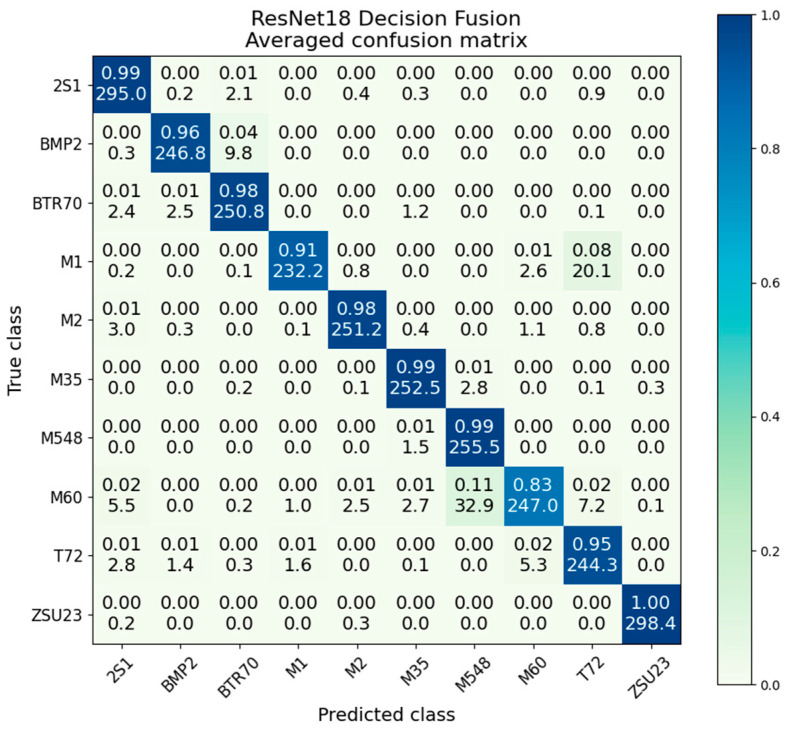
Averaged confusion matrix for ResNet18 decision fusion.

The confusion matrix for decision fusion is presented in Figure 14. Strong performance is noted in all classes, with significant improvement over both the SAR and EO confusion matrices. While the M1/T72 confusion is still present, its effects are significantly diminished to only 91%/8%. The worst performing M60 class scores 83% accuracy, compared to 87% in EO and 79% in SAR; this is the only class where there is a negative gain in performance compared to the best voting network.

The variances and standard deviation presented in Table 6 show that decision fusion has a very low intraclass variance, with an overall average of 5.2 × 10^−3^. The SAR- and EO-only ResNets have average variances of 3.2 × 10^−2^ and 2.8 × 10^−2^ respectively; this shows the significant improvement in consistency gained from the decision fusion method. We also note the M1/T72 class has a variance of 1.8 × 10^−2^, about half that of the EO-only ResNet at 3.9 × 10^−2^. This shows that despite the higher average accuracy, there is still some variation in the M1/T72 confusion from iteration to iteration.

**Figure 15:**
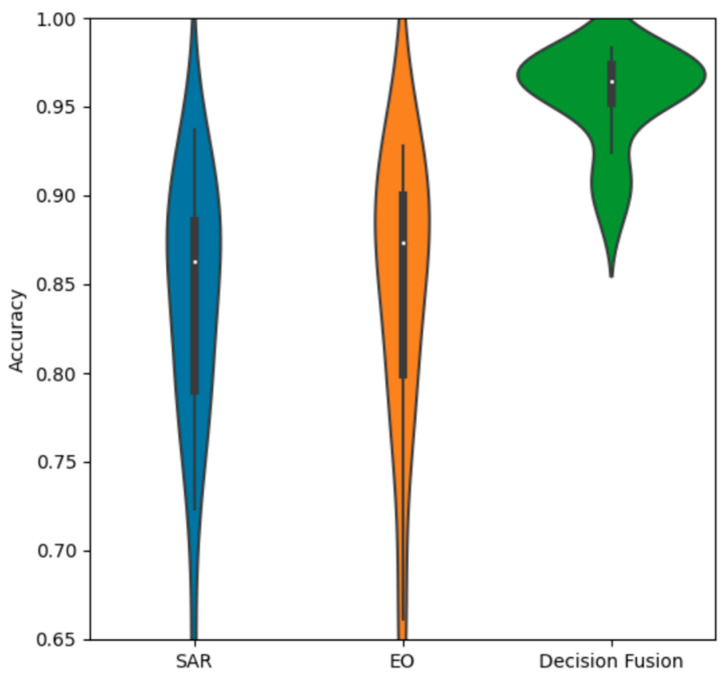
Accuracy distributions of decision fusion vs. SAR and EO.

The violin chart in Figure 15 compares the SAR and EO accuracy distributions to the decision fusion distribution. It is evident that decision fusion provides a significant gain in performance, and that it provides significantly more consistent accuracy over many trials. The main bell of the distribution is similar to a normal Gaussian distribution, with a tail of a few lower-performing outliers.

### 5.6. Simple Net Decision Fusion

**Figure 16:**
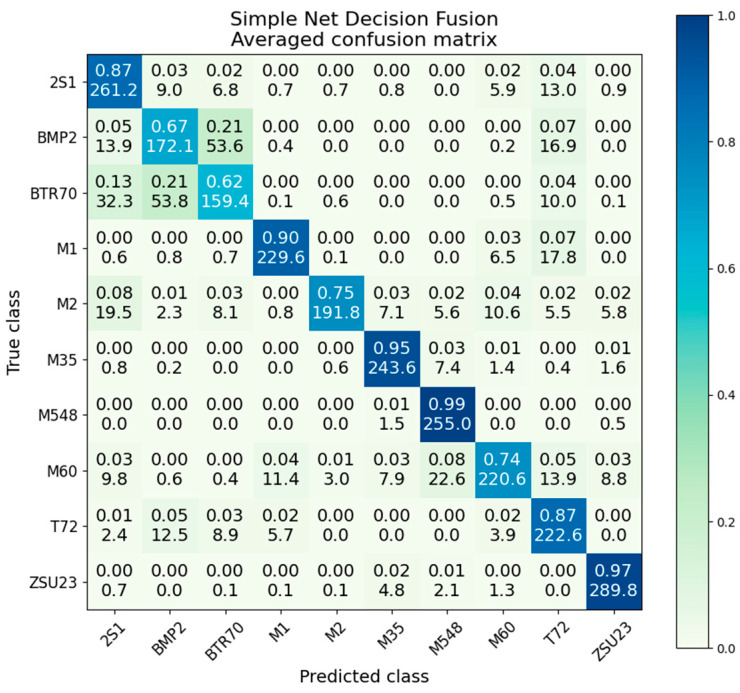
Averaged confusion matrix for simple net decision fusion.

Decision fusion with the results of the simple network in an accuracy of 83.4%, a gain of 11.6% over the better-performing SAR, and 15.2% over the worse−performing EO. The confusion matrix is given in Figure 16. While not as impressive of an accuracy as the ResNet decision fusion, it does manage to just outperform the individual modality ResNets. All classes experience a gain in performance over the best-performing voter, with the exception of the BTR70 and M60 classes which perform slightly better in the SAR-only network than in decision fusion. The particularly egregious T72 class, which had average accuracies of 68% and 26% in the EO and SAR voters, is significantly improved to 87% and a variance of only 7.9 × 10^−3^.

The intraclass variance of the simple net decision level fusion is given in Table 5, Table 6 and Table 7. The average variance is 1.2 × 10^−2^, compared to 5.2 × 10^−3^ by the ResNet decision fusion. This supports what was observed in the violin plots, where ResNet decision fusion produced significantly less variability and a tighter overall distribution. The average variance of 1.2 × 10^−2^ can be compared to the EO- and SAR-only simple nets, with variances of 2.02 × 10^−2^ and 2.45 × 10^−2^ respectively. We observe that there is still a variance reduction, albeit less of a dramatic reduction than seen in the ResNet case.

In this method, BMP2 is one of the highest variance classes at 1.7 × 10^−2^, whereas in ResNet decision fusion it is one of the lowest variance classes at 3.9 × 10^−4^. Similarly, M1 scores well here with a variance of 7.8 × 10^−3^ and an accuracy of 90%, but is the worst performing class in ResNet, with a variance of 2.2 × 10^−2^ and an accuracy of 91%. Even in this extreme case, the ResNets still outperform the simple nets, albeit with a slightly increased variance.

**Figure 17:**
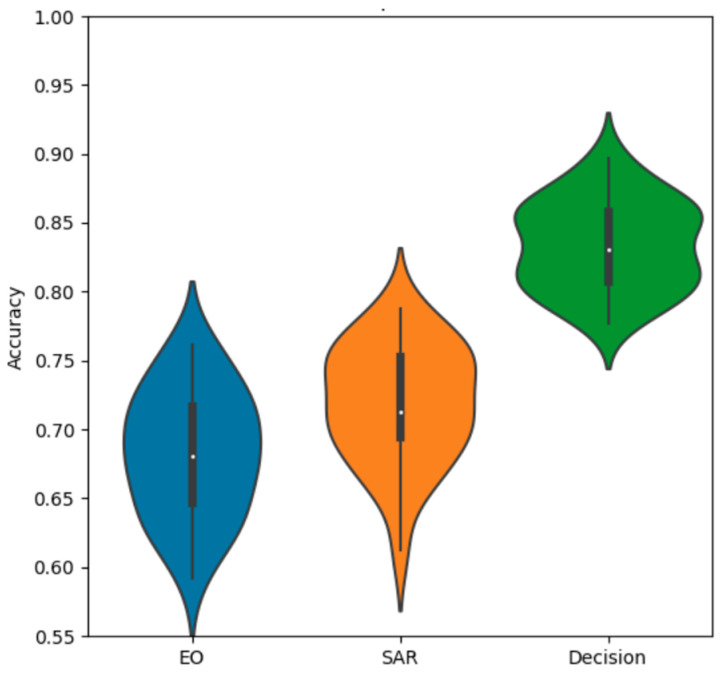
Accuracy distributions of simple net decision fusion vs. SAR and EO.

In the case of ResNet, the decision fusion had a significantly tighter accuracy distribution than the standalone modalities. However, the average was approaching the upper limit of 100% accuracy, meaning underlying variance may have been reduced. We observe the accuracy distribution of the simple network decision fusion in Figure 17. As the overall accuracy is reduced, we can more accurately observe the accuracy distribution of fusion. It is noted that the decision fusion still has a tighter distribution than the individual modalities, although the effect is somewhat reduced.

### 5.7. Simple Net Feature Level

**Figure 18:**
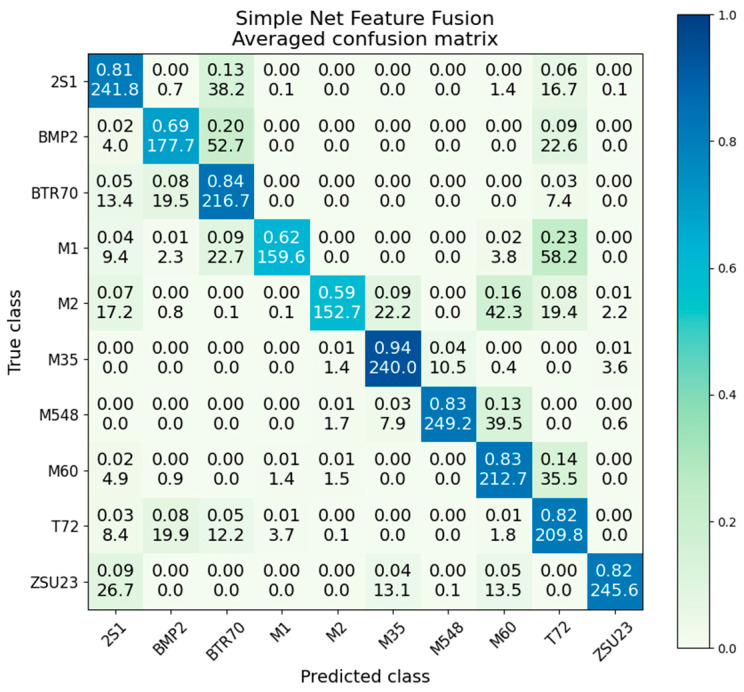
Averaged confusion matrix for simple net feature fusion.

Feature fusion using the simple network provided significant improvement up to 79.5% average accuracy over SAR (71.8%) and EO (68.2%) alone, although it fell short of the decision fusion scheme at 83.4%. The averaged confusion matrix is provided below in Figure 18. The highest-performing class achieves 94% accuracy, while the lowest achieves 59%. The recurring M1/T72 interclass confusion is present, with 23% of M1 images being misidentified to the T72.

The table of accuracies, variances, and standard deviations is also provided in Table 8. The average variance is 1.3 × 10^−2^, just slightly larger than simple net decision fusion at 1.2 × 10^−2^.

**Figure 19:**
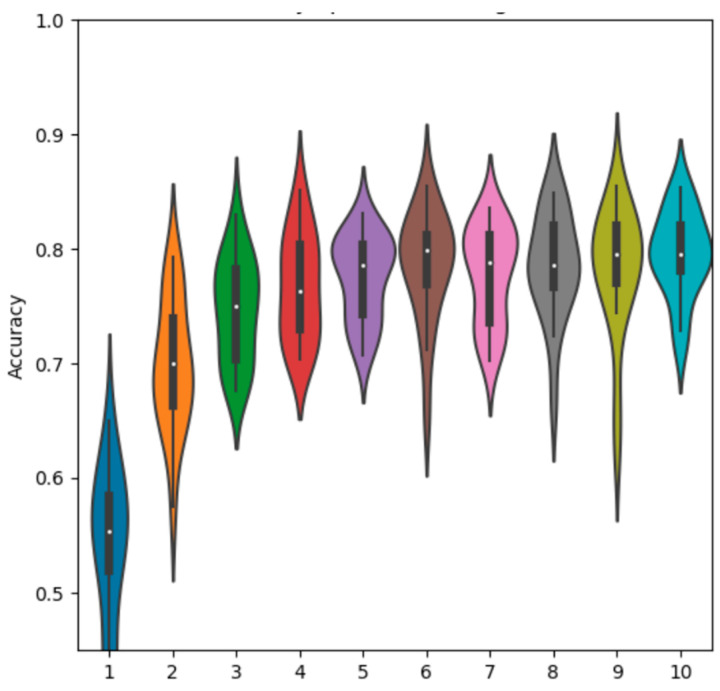
Accuracy distributions of simple net decision fusion vs. SAR and EO by epoch.

The testing accuracy distribution after each epoch of training is observed in Figure 19. Similar to the other simple network methods, we see a distinct rise in accuracy over the first few epochs which levels off in the second half of training. The networks also begin to for distributions similar to their final shape fairly early on, obviously with some slight variation. The central accuracy is fairly consistent across the final epochs.

### 5.8. ResNet18 Feature Level Fusion

**Figure 20:**
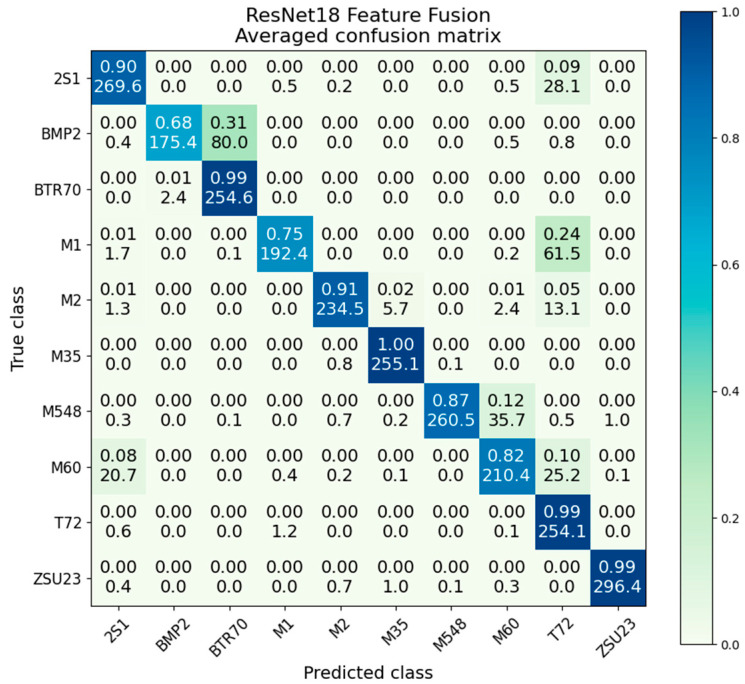
Averaged confusion matrix for ResNet18 feature fusion.

ResNet feature level fusion provided improvement beyond the individual modalities, although did not outperform decision fusion. Feature fusion provided an overall average classification accuracy of 90.4%, compared to 82.8% for EO-only and 80.3% for SAR-only. Similar to decision fusion, the distribution of accuracies with feature fusion is much more compact than SAR or EO. It also provides higher accuracy than the channel fusion presented in the next section. Figure 20 gives the confusion matrix.

**Figure 21:**
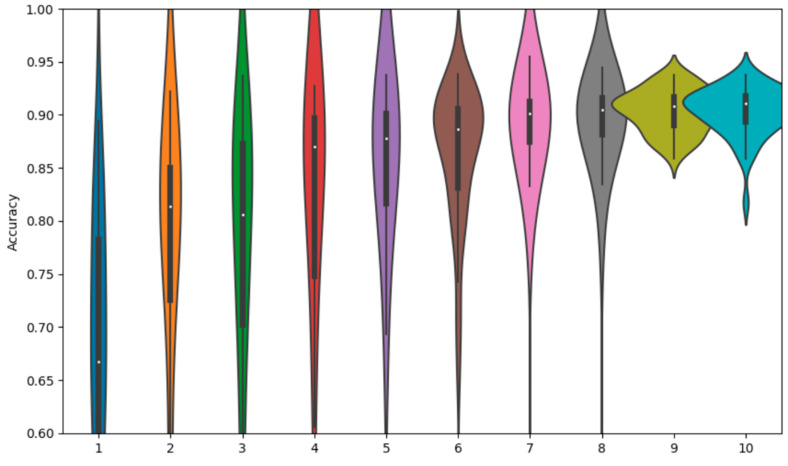
ResNet18 features fusion accuracy distributions during training by epoch.

The violin plot in Figure 21 shows the testing accuracy after each epoch of training. The pattern here is unique compared to the previously observed by-epoch patterns. Feature fusion starts with a very wide distribution and only begins to tighten somewhat after 5 or 6 epochs. Even then, it takes until the 9th and 10th epochs before the network rapidly converges to its final distribution shape. Comparing the variance values, given in Table 9, ResNet feature fusion scores an average variance of 3.4 × 10^−3^, the lowest of any of the fusion methods.

It is also worth noting that there is increased difficulty in performing feature-level fusion on a prebuilt network. Because the network’s layers have been predefined, they require more intense coding to access and mutate, for example when combining multiple parallel networks into a feature-level fusion scheme for multiple data types as performed here. By comparison, the simple custom-built network provides much easier access to its structure and layers, as it was custom programmed; however, this comes with a significant performance trade−off in overall classification accuracy.

**Figure 22:**
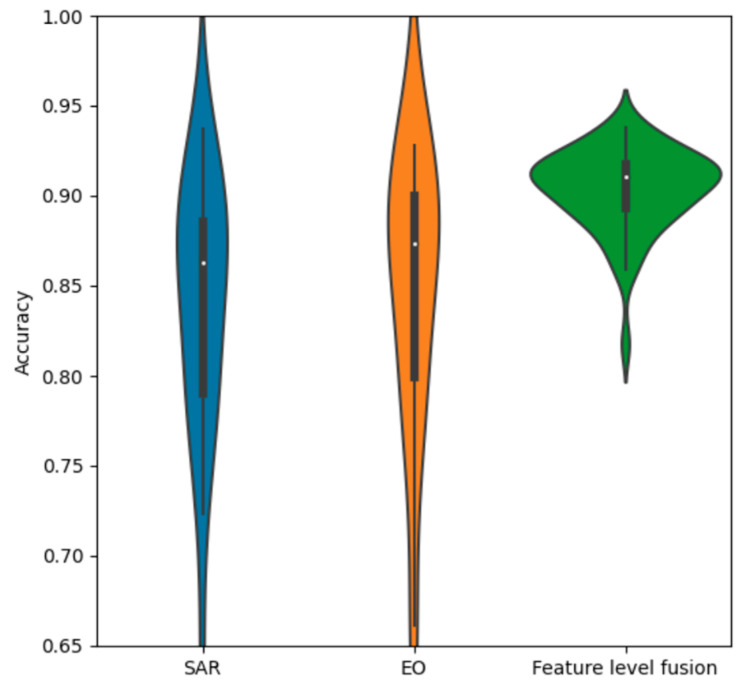
Accuracy distributions of ResNet18 feature fusion vs. SAR and EO.

Comparing the violin plots for SAR and EO in Figure 22 shows the substantial performance gain obtained by the feature-level fusion scheme. In addition to improving overall accuracy, feature fusion produces a much more compact distribution. The main bell of the distribution appears to be similar to a normal Gaussian distribution, with a very small number of lower outliers.

### 5.9. ResNet18 RGB Channel Fusion

**Figure 23:**
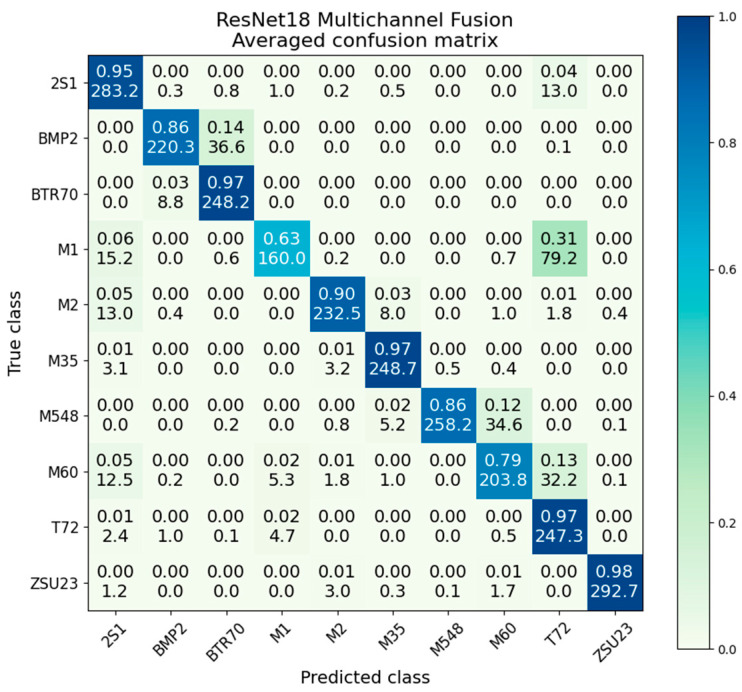
Averaged confusion matrix for ResNet18 RGB channel fusion.

With 88.9% classification accuracy overall, the channel fusion method provides surprisingly strong performance as simple as it is. Although it cannot achieve the accuracy of decision-level fusion, it is only 1.5% shy of feature fusion. The confusion matrix is provided in Figure 23. Feature fusion scores much better at 1.5 × 10^−3^, although channel fusion has a slightly lower M1/T72 variance than decision fusion at 1.8 × 10^−2^, as deduced from Table 10.

It is observed that the main performance degradation in the channel fusion method comes from a confusion between M1/T72, but the channel fusion has actually exacerbated that relationship. Compared to the EO-only, which had a 76%/15% split, channel fusion has a 63%/31% split. Except for that notable exception, most other classes are experiencing a suitable performance gain.

**Figure 24:**
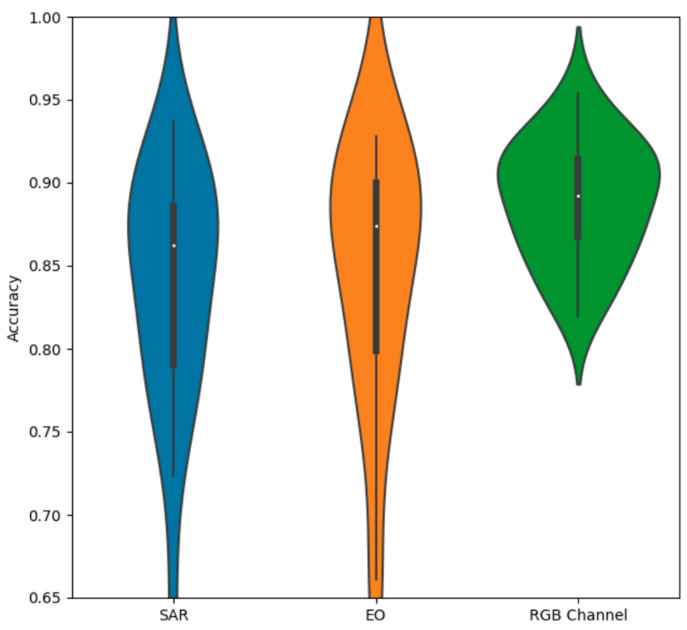
Accuracy distribution of RGB channel fusion vs. SAR and EO.

In line with its overall average accuracy being the lowest of the fusion methods, channel fusion also produces an accuracy distribution lower than a feature or decision-level fusion. We observe that the average intraclass variance has a value of 1.0 × 10^−2^, more than three times the variance of SAR-only (3.2 × 10^−2^) or EO-only (2.7 × 10^−2^) ResNets; a visual comparison of the distributions is given in Figure 24. However, its value is larger than that of decision fusion (5.2 × 10^−3^) and feature fusion (3.4 × 10^−3^), which matches our expectations from the violin plot of the distributions. We also note the value of the M1/T72 variance to be 9.2 × 10^−3^, an improvement over the EO-only variance of 3.9 × 10^−2^.

**Figure 25:**
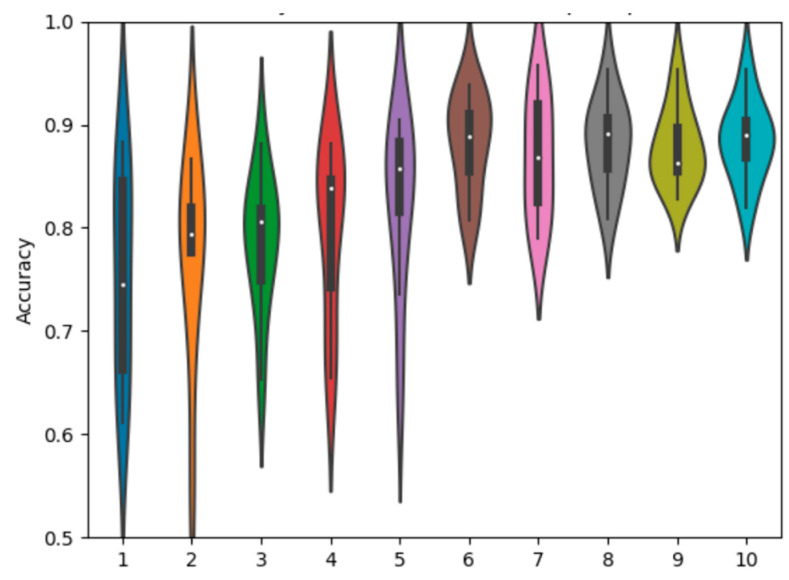
RGB channel fusion accuracy distributions during training by epoch.

Channel fusion produces an interesting pattern throughout its training, observed in Figure 25. The accuracy distributions for early epochs are very wide before quickly tightening towards their final distribution after the fifth epoch. While other methods follow a similar pattern of narrowing distributions as training goes on, the change in channel fusion is more abrupt. The number of outliers is significantly reduced beyond this point, where other methods tend to see them removed more linearly with increased training.

### 5.10. Network Sizes

While overall accuracy is important, another important aspect of a network is its size and computational intensity. A larger, more complicated network will require more memory, and more computational power to maintain the same rate of calculation; both of these may be important considerations when evaluating a fusion scheme’s viability in the field.

The total memory necessary to store each network may be compared. A standard standalone ResNet18 model has a size of 45.7 megabytes (MBs). The SAR and EO standalone networks have the same size of 45 MB, as do the channel fusion networks (channel fusion uses a single network processing both types of data via the three-color input channels meant for R-G-B images). By comparison, the ResNet18 feature fusion network has a size of 90.5 MB, almost twice that of a single ResNet18 model. This near doubling in size is expected, as the feature fusion is essentially two networks running in parallel for most of their structure, until the final feature level very near the end; hence almost the entirety of two networks’ worth of memory is needed. Similarly, decision fusion requires two separate SAR and EO networks, meaning it will require 91.5 MB of memory (two times the size of a standard ResNet18 model).

**Figure 26:**
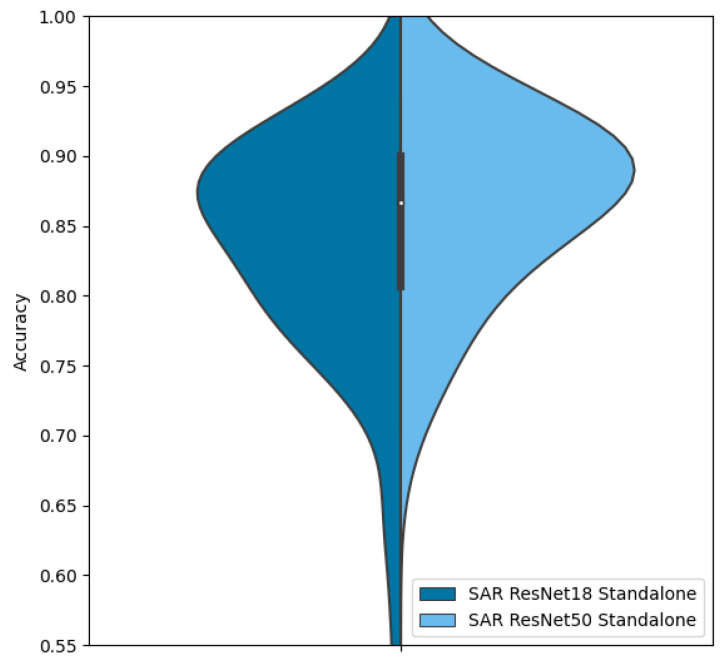
Accuracy distributions of ResNet18 vs. ResNet50 on SAR data.

By comparison, a ResNet50 model (the 50-layer equivalent to ResNet18) requires 100.1 MB of storage. Comparing the accuracy on SAR alone, we see an increase in accuracy to 85.5%, a gain of only 3% for a doubling in size over the ResNet18 model, visualized in Figure 26. Comparing the ResNet50 to feature fusion or decision fusion using ResNet18, both of which will require a similar 100 MB of storage, we note that the gain in accuracy is much greater for one of the fusion methods than increasing the size of the net.

Despite taking up the same amount of storage as the standalone networks, channel fusion improves the overall accuracy to 88.9% from 80.3% and 82.8%. While feature fusion performs slightly better at 90.4%, that additional 1.5% gain in accuracy comes at the cost of doubling our computation complexity. Similarly, in the case of decision fusion, which outperforms channel fusion by 6.5%, the performance gain comes at the cost of a doubling in required computation power.

By comparison, the custom-defined network used in the experiments is 23.497 MB in size, conveniently about half the size of a ResNet18 model and ¼ the size of a ResNet50 model. This advantage in storage size does not calculate savings in testing time, most likely due to the fact it is competing with an off-the-shelf model.

To compare the networks’ testing speed, we observed the elapsed time per testing image. Noting that elapsed times will depend on the computing power of the system, the relative value is most important. While one system may process two methods in 2 ms and 2.5 ms respectively, a slower system may process those methods in 4 ms and 5 ms, maintaining the same ratio of rates at a lower overall speed.

All of the calculation speeds were collected via Python’s “time” function, starting directly before testing was initiated and ending immediately after. This total time was used to calculate the average per frame. The speeds were collected for all iterations of a given experiment; while slight variations occurred between different iterations, all were very close to the final average. For both ResNet18 and the simple net, the additional computation of decision fusion in addition to the component networks adds 0.19 ms of calculation time per image. This gives a total of 1.29 + 1.88 + 0.19 = 3.36 ms per image for the ResNet, and 1.55 + 2.11 + 0.19 = 3.85 ms for the simple net.

Despite their smaller size, the simple nets take slightly longer on average to process than their ResNet counterparts; EO data also takes slightly longer to process than SAR. Comparing fusion methods, we see that they all operate in a similar time frame, about twice that of the standalone networks. For example, despite having the structure of a single network, the RGB channel fusion scheme still requires 3.33 ms to calculate an image, on par with decision and feature level fusion. So while channel fusion provides advantages in the size of the network and necessary storage, it does not provide advantages in computation time. This is a key distinction to note when evaluating the advantages and disadvantages of fusion methods for a particular application. A summary of the calculation times and network storage sizes is given in Table 11.

## 6. Conclusions

### 6.1. Discussion of Results

Within this paper, multiple data fusion methods for combining synthetic aperture radar and electrooptical data for classification with neural networks were compared: SAR and EO data individually, feature level fusion, decision level fusion, and RGB channel-based fusion. These methods were compared both with a custom-defined six-layer simple network and a more complicated off-the-shelf ResNet18, an 18-layer state-of-the-art three-channel image processing network.

Simple strategies and networks were used to examine the possibilities for performance gain that were available with each method. While networks and fusion methods exist which would produce a higher overall accuracy, it is difficult to evaluate a network’s performance as it converges towards near-perfect classification accuracies. The selected networks and fusion methods are well suited for the SAMPLE dataset, where a distribution of accuracies can be observed as many iterations of a specific experiment type (network size and fusion method) are trained.

Many iterations of each experiment type were run to study the inherent variability of the neural network training process. Distributions of the final accuracies in the form of violin plots, and averaged confusion matrices and variances were collected and observed. Testing accuracy was also recorded after every epoch of training to observe the training tendencies over many iterations.

The performance deficits between the simpler custom network and the prebuilt ResNet were observed to be significant for every fusion method explored. The deeper and more complicated ResNet was able to consistently perform to a higher classification accuracy in every equivalent scenario, without the added difficulty of constructing the layers from the ground up, as was necessary with the custom network, a process that requires more involvement and investment on the part of the designer.

While the out-of-the-box individual and decision-fused ResNets were easier to implement, as such implementations required no changing of the layer structure, the same cannot be said for the feature-level fusion. With the prebuilt nature of the ResNet models, fusing two parallel networks at their feature levels required significant coding to access their feature vectors and create the fusion scheme. The feature-level fusion experiments did provide an improvement in classification accuracy over a single-modality network alone.

By contrast, utilizing the RGB image color channels of the ResNet provided a competitive method to fuse SAR and EO data at an earlier feature level in the neural network. A small difference in final accuracy was noted, with the channel method slightly underperforming the bespoke method by 1.5% accuracy on average. It is also noted the distribution of channel fusion accuracies was wider than that of the bespoke fusion. The RGB channel fusion scheme offers an off-the-shelf implementation of fusion at a middle feature level, whereas the bespoke feature-level fusion scheme requires a dissection of the prebuilt networks to access their convolutional layers and assemblage of additional code. Similarly, it offers the opportunity to easily include a third sensor. Channel fusion also offered the advantage of smaller storage size, necessitating storage of only one network size as opposed to two sets of parallel layers.

Both the bespoke feature level and RGB-channel fusion methods suffered from underperformance on two classes, M1 and T72, of the dataset and fell just short of matching the performance of decision fusion using individual ResNets. Despite this, channel fusion offers strong enough results to warrant mention and future exploration, and could serve as a functional and simple method of implementing feature fusion with less involved coding and careful network construction required, as well as potential computational savings.

It was found that the average variance of the fusion methods was lower with the ResNet, with the exception of the standalone networks. The SAR-only and EO-only ResNets had high variance, but this was greatly reduced by the feature fusion and decision fusion schemes. The simple network fusion schemes did reduce its variance, but not as much as in the case of ResNet.

Given the conditions laid out by the methods of the paper, the ResNet decision fusion performed the “best,” as in achieving the highest overall average accuracy. However, there are several additional parameters discussed in the paper, such as in- and out-of-class confusion, consistency of convergence via the violin charts of accuracies, and so on. When coupled with the conditions of an outside “real-world” test, all of these considerations will result in a more satisfactory decision output.

The processing time of the methods investigated is discussed in Table 11 and the surrounding section. While there are a number of Python and PyTorch packages that claim to offer estimates of the number of FLOPs, their claims are unverifiable and can provide, at best, a rough estimate of the processing power necessary. The recorded processing time was used as a stand-in, as it seemed to provide the best analog to real-world ATR scenarios while providing a hard number for comparison. The calculated processing times were found to be very stable across multiple tests of the same trained models, regardless of other conditions on the system used for the calculation. This was sufficient evidence for us to trust and use the recorded processing times as a suitable metric to compare the computational intensity of a given network model and fusion method.

When discussing the complexity of the techniques, it should be noted that the complexity will be model dependent as well as parameter dependent. The effects of optimization are also noted, and it is worth pointing out again that the simple net takes slightly longer to calculate than the more optimized ResNets.

### 6.2. Avenues for Future Work

Potential avenues for future work include evaluating the performance of these fusion networks trained and tested on images from different azimuth and elevation angles; while this may provide the networks with a greater processing challenge with disparate data, it may also provide additional information that counteracts or overcomes this challenge to some degree. Another avenue would be to recreate the fusion methods for larger-sized ResNets; while the performance of ResNet50 was evaluated on SAR data, its performance on EO data and with the fusion methods was not pursued.

Another topic of exploration would be the comparison of these networks and methods on another similar dataset. As the results of machine learning are extremely reliant on the data used, recreating these methods with another two-modality dataset would provide useful insight into the nature of the methods. While few SAR and EO airborne datasets exist, there may be suitable SAR and EO satellite imagery datasets with similar properties. There are also multimodal imagery datasets available using electrooptical and LiDAR, and other sensor types common for self-driving vehicles. The general pattern of results is expected to be the same, but it could open up new subtleties to explore; a larger dataset could provide lower variance convergences, and a smaller dataset could provide a more distinctive distribution as networks become more specialized.

A subset of this could be achieved by training on smaller subsets of the SAMPLE dataset, although such an experiment would be ideal when starting with a larger dataset. With a larger starting dataset, more granularity in subset size could be explored without over-impacting training with too few images on the low end.

Another avenue would be pursuing more mixed signal-to-noise (SNR) datasets. Using data augmentation to supplement the already existing SAMPLE set with additional mixed SNR images would be a worthy avenue of continued work. As datasets with more sensors become more widely available, it would be interesting to continue this vein of work on different types of imagery beyond SAR and EO. Other satellite-based SAR and EO datasets, particularly those with a wider variety of targets, would also be interesting to examine. Finding sensors in further separated parts of the frequency spectrum would be similarly interesting, as well as including non-imagery sensors.

In our paper, the SNR of each SAR, and the corresponding synthetic EO image, are of the order of 18 dB. While the fusion technique provides a gain based on the same SNR inputs, the SNR of the SAR and the EO imagery may be different in real operating scenarios, e.g., a platform carrying both SAR and EO sensors. Therefore, the creation of synthetic EO images based on the SNR differences of SAR and EO, followed by an analysis of AI-based data fusion gains through CNN and ResNet-based training and classification is an interesting topic for further research.

The effects of the size of the final classification vector are also of interest. While the SAMPLE dataset only contains ten targets, a dataset with a wider variety of targets could be used to test the networks’ abilities to adapt to broader classification problems. It is likely a larger network such as the ResNet would be able to handle a higher class count more easily, but the structure of the final layers of the network may come into play even more when examining wider classification problems.

Since the body of work available on machine learning models and fusion methods is expanding rapidly, another area of future work is the exploration of state-of-the-art techniques in both aspects. In addition, joint or shared learning approaches, especially as they apply to multimodal sensor data, need to be investigated.

An interesting avenue for future consideration would be within a single accuracy distribution for a particular method, what the processing times were for different models across the distribution. Only the average frame time across all models of a particular type was explicitly presented, but examining the calculation time per frame for each individual instance would be worth investigating

It is noted in the paper that the distribution of calculation times across iterations is rather tight; that is, there are much higher variance inaccuracies than in calculation times. Examining the possible correlations between those metrics would be of interest, but due to little difference in calculation times observed within instances of a particular experiment type, it was not deemed a useful exploration for this initial piece of work.

## Figures and Tables

**Table 1:** Average overall classification accuracies of each fusion method.

Experiment	Simple Net	ResNet18
SAR Standalone	71.8%	82.8%
EO Standalone	68.2%	80.3%
Decision Level Fusion	83.4%	95.6%
Feature Level Fusion	79.5%	90.4%
RGB Channel Fusion	-	88.9%

**Table 2:** SAR-only ResNet18 intraclass variance (Var) and standard deviation (SD).

Class	2S1	M60	BMP	M2	T72	M1	BTR	M548	M35	ZSU
Mean	77%	79%	80%	72%	76%	81%	84%	96%	91%	94%
Var	7.1 × 10^−2^	5.9 × 10^−2^	4.3 × 10^−2^	3.8 × 10^−2^	3.5 × 10^−2^	3.0 × 10^−2^	2.0 × 10^−2^	9.9 × 10^−3^	8.2 × 10^−3^	5.4 × 10^−3^
SD	2.7 × 10^−1^	2.4 × 10^−1^	2.1 × 10^−1^	2.0 × 10^−1^	1.9 × 10^−1^	1.7 × 10^−1^	1.4 × 10^−1^	1.0 × 10^−1^	9.0 × 10^−2^	7.0 × 10^−2^

**Table 3:** SAR-only simple net intraclass variance (Var) and standard deviation (SD).

Class	T72	BTR	BMP	M1	M2	M60	2S1	M35	M548	ZSU
Mean	26%	65%	55%	65%	59%	78%	84%	82%	97%	96%
Var	6.7 × 10^−2^	5.7 × 10^−2^	3.3 × 10^−2^	2.6 × 10^−2^	2.6 × 10^−2^	1.6 × 10^−2^	8.6 × 10^−3^	8.0 × 10^−3^	2.2 × 10^−3^	1.6 × 10^−3^
SD	2.6 × 10^−1^	2.4 × 10^−1^	1.8 × 10^−1^	1.6 × 10^−1^	1.6 × 10^−1^	1.2 × 10^−1^	9.0 × 10^−2^	9.0 × 10^−2^	5.0 × 10^−2^	4.0 × 10^−2^

**Table 4:** EO-only ResNet18 intraclass variance (Var) and standard deviation (SD).

Class	M1	T72	BMP	M60	BTR	M35	M2	M548	2S1	ZSU
Mean	76%	85%	82%	87%	93%	92%	89%	96%	90%	99%
Var	6.0 × 10^−2^	4.7 × 10^−2^	4.2 × 10^−2^	3.5 × 10^−2^	2.7 × 10^−2^	2.3 × 10^−2^	1.9 × 10^−2^	1.2 × 10^−2^	8.5 × 10^−3^	1.5 × 10^−3^
SD	2.4 × 10^−1^	2.2 × 10^−1^	2.0 × 10^−1^	1.9 × 10^−1^	1.6 × 10^−1^	1.5 × 10^−1^	1.4 × 10^−1^	1.1 × 10^−1^	9.0 × 10^−2^	4.0 × 10^−2^

**Table 5:** EO-only simple net intraclass variance (Var) and standard deviation (SD).

Class	BMP	M2	BTR	M60	M1	2S1	ZSU	T72	M35	M548
Mean	54%	55%	44%	58%	73%	71%	82%	68%	80%	91%
Var	3.3 × 10^−2^	3.2 × 10^−2^	2.8 × 10^−2^	2.6 × 10^−2^	2.1 × 10^−2^	2.0 × 10^−2^	1.5 × 10^−2^	1.3 × 10^−2^	1.0 × 10^−2^	4.4 × 10^−3^
SD	1.8 × 10^−1^	1.8 × 10^−1^	1.7 × 10^−1^	1.6 × 10^−1^	1.5 × 10^−1^	1.4 × 10^−1^	1.2 × 10^−1^	1.1 × 10^−1^	1.0 × 10^−1^	7.0 × 10^−2^

**Table 6:** ResNet18 decision fusion intraclass variance (Var) and standard deviation (SD).

Class	M1	M60	T72	BTR	M2	M548	2S1	M35	BMP2	ZSU
Mean	91%	83%	95%	98%	98%	99%	99%	99%	96%	100%
Var	2.2 × 10^−2^	1.1 × 10^−2^	1.1 × 10^−2^	4.5 × 10^−3^	1.1 × 10^−3^	7.2 × 10^−4^	6.8 × 10^−4^	4.1 × 10^−4^	3.9 × 10^−4^	5.0 × 10^−5^
SD	1.5 × 10^−1^	1.1 × 10^−1^	1.0 × 10^−1^	7.0 × 10^−2^	3.0 × 10^−2^	3.0 × 10^−2^	3.0 × 10^−2^	2.0 × 10^−2^	6.0 × 10^−2^	1.0 × 10^−2^

**Table 7:** Simple net decision fusion intraclass variance (Var) and standard deviation (SD).

Class	BTR	M2	BMP	M60	2S1	T72	M1	M35	ZSU	M548
Mean	62%	75%	67%	74%	87%	87%	90%	95%	97%	99%
Var	3.0 × 10^−2^	2.7 × 10^−2^	1.7 × 10^−2^	1.3 × 10^−2^	9.7 × 10^−3^	7.9 × 10^−3^	7.8 × 10^−3^	3.7 × 10^−3^	3.4 × 10^−3^	2.7 × 10^−4^
SD	1.7 × 10^−1^	1.6 × 10^−1^	1.3 × 10^−1^	1.1 × 10^−1^	1.0 × 10^−1^	9.0 × 10^−2^	9.0 × 10^−2^	6.0 × 10^−2^	6.0 × 10^−2^	2.0 × 10^−3^

**Table 8:** Simple net feature fusion intraclass variance (Var) and standard deviation (SD).

Class	ZSU	BMP	T72	M1	M2	BTR	M60	M548	M35	2S1
Mean	82%	69%	82%	62%	59%	84%	83%	83%	94%	81%
Var	2.7 × 10^−2^	2.5 × 10^−2^	1.8 × 10^−2^	1.5 × 10^−2^	1.4 × 10^−2^	1.2 × 10^−2^	9.2 × 10^−3^	7.2 × 10^−3^	1.8 × 10^−3^	1.8 × 10^−3^
SD	1.6 × 10^−1^	1.6 × 10^−1^	1.3 × 10^−2^	1.2 × 10^−1^	1.2 × 10^−1^	1.1 × 10^−1^	1.0 × 10^−1^	9.0 × 10^−2^	4.0 × 10^−2^	4.0 × 10^−2^

**Table 9:** ResNet18 feature fusion intraclass variance (Var) and standard deviation (SD).

Class	BMP	M2	2S1	M60	M1	M548	BTR	T72	ZSU	M35
Mean	68%	91%	90%	82%	75%	87%	99%	99%	99%	100%
Var	8.5 × 10^−3^	7.6 × 10^−3^	7.0 × 10^−3^	6.9 × 10^−3^	1.5 × 10^−3^	1.3 × 10^−3^	4.0 × 10^−4^	3.8 × 10^−4^	3.6 × 10^−4^	2.7 × 10^−4^
SD	9.0 × 10^−2^	9.0 × 10^−2^	8.0 × 10^−2^	8.0 × 10^−2^	4.0 × 10^−2^	1.1 × 10^−1^	2.0 × 10^−2^	9.0 × 10^−2^	2.0 × 10^−2^	2.0 × 10^−2^

**Table 10:** RGB channel fusion intraclass variance (Var) and standard deviation (SD).

Class	M60	BMP	M1	M2	M548	M35	2S1	T72	BTR	ZSU
Mean	79%	86%	63%	90%	86%	97%	95%	97%	97%	98%
Var	3.9 × 10^−2^	2.1 × 10^−2^	1.7 × 10^−2^	8.4 × 10^−3^	3.8 × 10^−3^	3.8 × 10^−3^	3.1 × 10^−3^	2.5 × 10^−3^	2.1 × 10^−3^	1.6 × 10^−3^
SD	2.0 × 10^−1^	1.5 × 10^−1^	1.3 × 10^−1^	9.0 × 10^−2^	6.0 × 10^−2^	6.0 × 10^−2^	6.0 × 10^−2^	5.0 × 10^−2^	5.0 × 10^−2^	4.0 × 10^−2^

**Table 11:** Calculation times and network storage sizes.

Experiment	Simple Net	ResNet18
SAR Standalone	1.55 ms/image	1.29 ms/image
	24 MB	45 MB
EO Standalone	2.11 ms/image	1.88 ms/image
	24 MB	45 MB
Decision Level Fusion	3.85 ms/image	3.36 ms/image
	50 MB	90 MB
Feature Level Fusion	3.66 ms/image	3.28 ms/image
	50 MB	90 MB
RGB Channel Fusion	-	3.33 ms/image
	-	45 MB

## Data Availability

No new data were created or analyzed in this study. Data sharing is not applicable to this article.
